# A mouse model of non-union in a femur critical-size defect using locking plate fixation

**DOI:** 10.1186/s13018-026-07099-2

**Published:** 2026-07-23

**Authors:** Thomas J. Kremen, Jeremy J. Reid, Kristine D. Estrada, Nicholas M. Bernthal, Timothy P. Liu, Al-Hassan J. Dajani, Karen M. Lyons

**Affiliations:** 1 Virtua Reconstructive Orthopedics, Virtua Health, Voorhees, NJ USA; 2 Department of Orthopaedic Surgery, David Geffen School of Medicine at UCLA UCLA, 10833 Le Conte Ave., CHS 76-143, Los Angeles, CA 90095 USA

**Keywords:** Nonunion, Fracture, Preclinical, Mouse, In vivo, Model

## Abstract

**Background:**

Mouse fracture models are powerful tools for investigating in vivo bone healing in response to specific genetic alterations or cellular and protein-based osteogenic therapies. However, there are a paucity of murine models that accurately and humanely recapitulate human fracture fixation, with the modelling of non-union posing a particularly difficult challenge. The objective of this study was to develop a translationally relevant non-union fracture model in mice.

**Methods:**

Twenty-six mice received a unilateral 1.75 mm femoral bony defect, while four control animals underwent a 0.25 mm osteotomy. Internal fixation of bony defects was performed using titanium locking plates and screws, and mice underwent weekly radiographic assessments postoperatively. Of the 1.75 mm osteotomy group, two animals were sacrificed at 5 days, four at 2 weeks, seven at 6 weeks, and thirteen at 12 weeks postoperatively. Histologic analysis was performed with Safranin O staining and immunohistochemistry for collagen types I and II. Nonunion was evaluated in the 6 and 12 week animals only (*n* = 20) to allow sufficient time for potential bony healing.

**Results:**

All control animals with 0.25 mm osteotomy demonstrated bony union of the femur by 2 weeks. In contrast, among the experimental group with 1.75 mm defects, 65% (13 of 20) of animals overall exhibited non-union. When stratified by postoperative healing time, 71% (5 of 7) of animals followed for 6 weeks showed non-union of the femur, whereas 62% (8 of 13) of the animals followed for 12 weeks showed non-union.

**Conclusions:**

This paper describes a translationally-relevant mouse model of long bone non-union that can be used to recapitulate the key clinical challenges and biology of human fractures. This valuable preclinical investigation technique can subsequently be used to study the effects of genome alterations as well as novel osteogenic scaffolds, drugs, and biologic therapies.

**Supplementary Information:**

The online version contains supplementary material available at https://doi.org/10.1186/s13018-026-07099-2.

## Background

While most fractured bones heal predictably, 5 to 10% of the millions of fractures that occur each year exhibit delayed or impaired healing, resulting in approximately 100,000 non-unions annually [1–3]. This problem has driven the need for more effective strategies to augment bony healing, especially in the settings of osteoporosis, non-unions, and bone deficits as a result of trauma [4–11]. Advances in implant design, such as locked plating constructs, have improved the mechanical properties of fixation techniques especially when absolute stability is not possible. Nevertheless, the long-term outcome of fractures is still ultimately dependent on biologic healing. Traditional surgical strategies aimed at improving the biology of bone healing have consisted primarily of autogenous or allogeneic bone grafting techniques [2, 5, 12–16]. More recently, experimental biologic enhancement strategies have included supplementation with growth factors either locally or systemically [4, 7, 17–22]. However, each of these modalities carries significant comorbidity, risk, and/or significant financial burden [4, 23–25]. As a result there continues to be a growing interest in biologic therapeutics, such as the delivery of genetically altered host cells capable of improving the osteogenic healing response [4, 26–31].

Given their well-defined and mutable genomes, mouse models have provided one of the best and most consistent approaches for investigating the effects of genetic engineering in vivo [30, 32]. Moreover, the mouse is the most common and versatile model in which it is possible to investigate the impact of specific genes or genetic backgrounds on the fracture healing process. Several mouse models have been described for studying fracture repair [33–35]. Intramedullary fixation models are commonly used and involve straight pin stabilization with variable addition of a pin to improve rotational stability [20, 36]. Internal plating systems with or without cement spacers are also used to evaluate bone defects of specific sizes [37]. Tay et al. described a ring external fixator to study long-bone fracture healing in a distraction osteogenesis model [33]. Similar external fixator devices were described by Sandberg et al. using a stable ex-fix after 0.2 mm osteotomy in mice [38] and by Carvalho et al. to study distraction osteogenesis via an external device fastened to a mouse tibia with cerclage wires [34, 39]. Hu et al. also used an external fixation system and described a method employing both osteotomy and distraction osteogenesis to create a non-union model [40]. Investigators have even described murine models of long-bone healing without fixation [35, 41]. For example, to evaluate cancellous bone healing in mice, Sandberg et al. described a model which evaluated the pullout strength of a tibial metaphyseal screw to evaluate the extent of bone healing [38, 42].

While these models have been very useful for understanding the biology of fracture repair, each of them has some limitations [43]. In some cases, it is not possible to create defects of defined sizes with accuracy, which complicates the interpretation of healing capacity. In other cases, the fixation devices impair mobility and thus impose significant morbidity on the study animals. In fracture models that do permit normal host ambulation, the murine healing response is so robust that detecting the efficacy of an intervention can be challenging [20, 44, 45]. Additional challenges arise when the fixation devices themselves can disrupt the biology of fracture healing, reducing clinical translatability. For example, the use of cerclage wire fixation can impair periosteal blood supply and as a result injure the cells of the periosteum. This is problematic given that the periosteum is a major source of mesenchymal stem cells that contribute to bony healing [6, 41, 46, 47]. Importantly, although animals such as mice often heal much faster than humans [48], they must be provided sufficient healing time to distinguish if they exhibit delayed union versus true nonunion. For example, mice have been reported to achieve bony bridging and remodeling of femur fractures by 3 weeks [49]. As a result, prior studies have used 6 or even 12 weeks as the cutoff for nonunion to allow for adequate recovery time and reduce potential mislabeling [50–52]. The effect of unpredictable interfragmentary strain levels between individual animals can also make the interpretation of results even more difficult [53, 54]. Moderate interfragmentary micromotion promotes endochondral ossification while rigid fixation with minimal strain promotes intramembranous healing [55, 56]. Endochondral ossification is a relatively common healing model that involves callus and cartilage formation with subsequent mineralization. Delayed union can therefore be characterized by persistent bridging cartilage/callus or incomplete ossification. On the other hand, there is a paucity of intramembranous healing models [20, 33, 43, 44, 57], which involve direct deposition of bone by osteoblasts with minimal cartilage formation. This can further complicate the interpretation of delayed union versus nonunion. Given the exceedingly common use of low strain and stiff internal fixation constructs, such as locked plating in contemporary fracture management, mouse models exhibiting intramembranous healing and utilizing locked plating may present a higher degree of clinical relevance.

The ability to heal a critically sized long-bone defect is a robust biological challenge that provides an excellent assay for preclinical investigations of osteogenic therapies. Therefore, the purpose of this investigation was to establish a critical-sized mouse femoral defect model using surgical techniques and fixation that accurately recapitulate the treatment of long bone fractures in humans.

## Methods

### Animals

Female C57BL/6J mice (Jackson Laboratory, Maine, USA) aged 10 to 14 weeks were maintained in a 12 h light and dark cycle and fed in a standard vivarium environment with water *ad libitum*. A total of thirty mice underwent surgery and were randomized to groups to reduce surgical order confounding in our IACUC approved research protocol complying with National Institute of Health guidelines. This study was carried out in accordance with the ARRIVE 2.0 Essential 10 guidelines. Twenty-six animals received a unilateral 1.75 mm femoral osteotomy; two were euthanized on post-operative day 5, four were euthanized on post-operative day 14, seven on post-operative day 42 and thirteen on post-operative day 84. Four animals received a 0.25 mm osteotomy and were euthanized at 6 weeks post-operatively to serve as control animals replicating previously published data [57]. The sample sizes for the 0.25 mm and 1.75 mm osteotomy groups (at 12 weeks postop) were calculated using power analysis with the following parameters: anticipated 75% nonunion incidence, 3:1 enrollment ratio, alpha of 0.05, and 95% power, with an additional animal added to the experimental 1.75 mm group in case of attrition. The 1.75 mm osteotomy group at 6 weeks postop followed the same power analysis and control group size with a 2:1 enrollment ratio.

The evaluation timepoints were chosen based on prior nonunion studies in mouse long bones and to allow for sufficient recovery time. Mice heal fractures significantly faster than humans, with a reported half-healing time of 12 days in mice versus 91 days in humans [48]. Prior data also shows that mouse femur fractures typically form callus by 2 weeks and progress to bony bridging and remodeling by 3 weeks [49]. Therefore, 12 weeks is substantially longer than the usual healing window, allowing sufficient time for the declaration of nonunion and reducing the likelihood of mislabeling delayed unions. Multiple prior studies of critical size femur defects in transgenic mice have also used 12 weeks as the terminal timepoint [50, 51]. Earlier cutoffs at 6 weeks are occasionally used, although these may introduce false positives through delayed union [52]. Lastly, prior mouse models of tibia fracture have shown minimal radiographic changes from 6 to 12 weeks, demonstrating a plateau in bony remodeling and healing [40] and suggesting that nonunion may be less likely to progress past this time.

### Implants

**Fig. 1:**
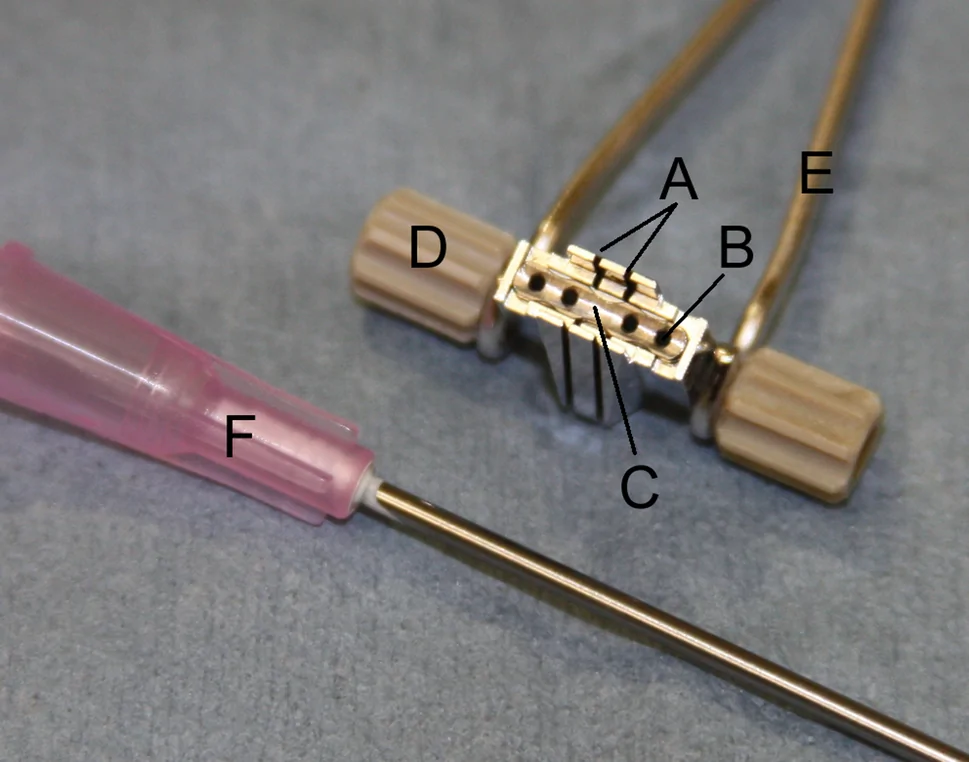
Internal fixation using the MouseFix™ system and a custom drilling guide. **A** Grooves in drill guide to accommodate Gigli saw (1.75 mm apart). **B** Guide holes for hand drill and screws. **C** Recess in drill guide to accommodate locking plate. **D** Ceramic nut to affix **E** drill guide to stabilization hand piece. **F** 18-guage hypodermic needle for relative size comparison

Femoral internal fixation was performed utilizing the commercially available MouseFix™ implant system (AO foundation, Research Implant Systems, Davos, Switzerland), consisting of a titanium locking plate with four locking screws. A defined osteotomy was reproducibly performed using miniature Gigli saws in conjunction with a custom-built drilling and cutting guide designed specifically for use with the MouseFix™ femoral locking plate and screws. Separate drilling guides were manufactured to enable the creation of 0.25 mm and 1.75 mm defects (Fig. 1). Osteotomy reproducibility was confirmed by individually measuring the resultant bone dowel excised after plate fixation and Gigli saw treatment. The 1.75 mm defect size served as the largest osteotomy generatable using the custom cutting guide. Briefly, the MouseFix™ plate is a commercially available system that is designed for the typical 14 mm length of an adult mouse femur. A custom drilling and cutting guide was designed based on the plate dimensions, and the largest osteotomy cut feasible using this system was 1.75 mm. Notably, this defect is smaller than previously-reported critical size defects using other fixation methods including intramedullary, plate, and external fixation [58–60].

### Surgical procedure

Mice were anesthetized with inhaled isoflurane mixed with 100% oxygen delivered via nose cone. Perioperative analgesia consisted of buprenorphine at a dose of 0.1 mg/kg and was administered subcutaneously after induction of anesthesia. Surgery was performed with sterile instruments utilizing standard sterile technique, with mice maintained in a prone position on a water-recirculating warming pad. The left femur of each animal was exposed using a lateral approach to the femur. The plate and drill guide were placed on the femur using submuscular technique, followed by drilling of the four drill holes. All drilled holes were filled with screws prior to making two separate osteotomies using two individual Gigli saws embedded into the pre-machined grooves of the osteotomy/drilling guide. After completion of the osteotomy, the resultant boney dowel was measured and the guide was removed from the plate. The wound was irrigated and closed in two layers using 5.0 DEXON™ suture (United States Surgical, North Haven, CT) with a lateral thigh muscle fascia layer followed by a buried subcuticular/deep dermal layer. DERMABOND™(Ethicon, Inc, Cornelia, GA) was applied to the entire incision and the animals were awoken and monitored for ambulatory status and signs of distress. Animals were administered buprenorphine 0.1 mg/kg subcutaneously every 12 h for 48 h total post-operatively. Sulfamethoxazole/Trimethoprim (Hi-Tech Pharmacal Co. Inc., Amityville, NY) supplemented water was administered for 1 week post-operatively at a dose of 2 mg/ml sulfamethoxazole and 0.04 mg/ml trimethoprim. Pre-operative and daily post-operative animal weights were measured and ranged from 19 g to 23 g with no animal weight loss noted post-operatively. All animals were ambulatory and used their operative limb for weight bearing by post-operative day 0. No animals were euthanized prematurely.

### Radiographic analysis

Images were obtained utilizing a Faxitron^®^ LX-60 Specimen Radiography System and SR Imaging Software with ImageAssist™ (Faxitron X-Ray LLC, Lincolnshire, IL, USA). Successive images consisting of two orthogonal views of each mouse femur were obtained immediately post-operatively as well as at weekly intervals thereafter until the time of euthanasia.

#### Histology and immunofluorescence staining

After resection of operative femurs and the surrounding soft tissue envelope at 2, 6 and 12 weeks post-operatively, the implants were removed and samples were fixed with 4% paraformaldehyde for 24 h, decalcified using Fast DeCal™ overnight and then paraffin embedded using standard protocols. Longitudinal 10 μm tissue sections were then stained with Safranin-O and Fast Green as previously described [61]. For immunofluorescence staining, antigen retrieval was performed by incubating sections in 1 mg/ml hyaluronidase (Sigma-Aldrich) in phosphate-buffered saline (Mediatech, Inc.) for 1 h at 37 °C. For detection of types I and II collagen (Abcam, ab21291), sections were blocked with 0.5% blocking reagent (TSA™ Biotin System, Perkin Elmer) in Tris-buffered saline (100 mM Tris pH 7.5, 150 mM NaCl), incubated with primary antibodies overnight at 4 °C, incubated with AlexaFluor-555 (Invitrogen) secondary antibodies for 30 min at room temperature, and counterstained with DAPI (Invitrogen). Samples were examined and images were obtained using an Olympus Microscope.

#### Fracture union assessment

Union or non-union of the femoral osteotomy site was defined by satisfying both radiographic and histologic criteria and was assessed by three independent observers, who were blinded to experimental groups and other observer’s assessments. Disagreements were resolved by majority decision (i.e. 2/3 assessments). Radiographic criteria for union were defined as evidence of cortical bridging (unicortical or bicortical) on at least one of two orthogonal views. This criteria was based on the findings of Disilvio et al., who reported that 1/4 bridging cortices were sufficient to predict union in > 95% of patients with femur fractures fixed with locked plating [62]. Histologic criteria for union were defined as the presence of bridging cortical bone or osteoid at the femoral osteotomy site on final histology. Non-union was therefore defined as the absence of the above radiographic and histologic criteria at 6 or 12 weeks after osteotomy (total *n* = 20 animals), with 6 and 12 weeks deemed as sufficient time to declare nonunion based on previous studies as described above in the “Animals” section. Two independent assessments of femoral osteotomy site union based on histological and radiological data were performed for each study animal (Supplemental Tables 1, 2, 3 and 4).

### Statistical analysis

Fisher’s exact test was used to assess for a difference in the rate of non-union among animals in the 0.25 mm osteotomy group compared to the 1.75 mm osteotomy group, as well as between animals euthanized at 6 weeks post-operatively compared to animals euthanized 12 weeks post-operatively. Fisher’s exact test was used given small sample sizes in categorical variables.

## Results

### Complications

All animals were weight bearing on the operative limb within minutes of awakening from anesthesia. None of the operative animals exhibited signs of distress (i.e. vocalization, weight loss, poor grooming, inactivity, infection, poor socialization) throughout the course of the experiments. There were no post-operative infections, periprosthetic fractures or hardware failures. Loss of fixation at the distal screw occurred in one instance, and this animal was thus excluded from analysis. The mean measured defect size was 1.75 mm with a range of 1.71 to 1.78 and a standard deviation of 0.02.

### Non-union rate

All animals (*n* = 4) that received a 0.25 mm femoral osteotomy demonstrated radiographic signs of union by 2 weeks, and none showed histologic evidence of non-union on final histology (Table 1, see Supplemental Table 1 for observer ratings). These findings are concordant with previously published data [57]. In contrast, the mice that underwent a 1.75 mm osteotomy and were sacrificed at 2 weeks post-operatively demonstrated no radiographic or histologic healing (*n* = 4, Table 1, observer ratings in Supplemental Table 2). In the 1.75 mm osteotomy group that was sacrificed at six weeks postoperatively, five of seven (71%) animals showed non-union (Supplemental Table 3). In the 1.75 mm osteotomy group after 12 weeks, eight of thirteen (62%) animals showed non-union (Supplemental Table 4). In total, thirteen of twenty animals at the 6 and 12 week timepoints (65%) exhibited non-union (Table 1). There was a significantly higher rate of nonunion in the 1.75 mm osteotomy group compared to the 0.25 mm osteotomy group (*p* = 0.03), and there was no statistically significant difference in the rate of non-union among the animals sacrificed at 6 weeks or 12 weeks (*p* = 1.00).

**Table 1:** Union and Nonunion Rates in 0.25 mm osteotomy control group and 1.75 mm osteotomy group at 6 and 12 weeks after surgery. *Data also reported for 1.75 mm group at 2 weeks after surgery, but not included in overall nonunion analysis due to insufficient healing time

Group (total *n*)	Animals with union (*n*)	Animals with no union (*n*)
0.25 mm Osteotomy (*n* = 4)	4	0
1.75 mm Osteotomy 6 weeks postop (*n* = 7)	2	5
1.75 mm Osteotomy 12 weeks postop (*n* = 13)	5	8
Overall 1.75 mm Osteotomy (6 and 12 weeks postop) (*n* = 20)	7	13
*1.75 mm Osteotomy 2 weeks postop (*n* = 4)	*0*	*4*

### Radiography

**Fig. 2:**
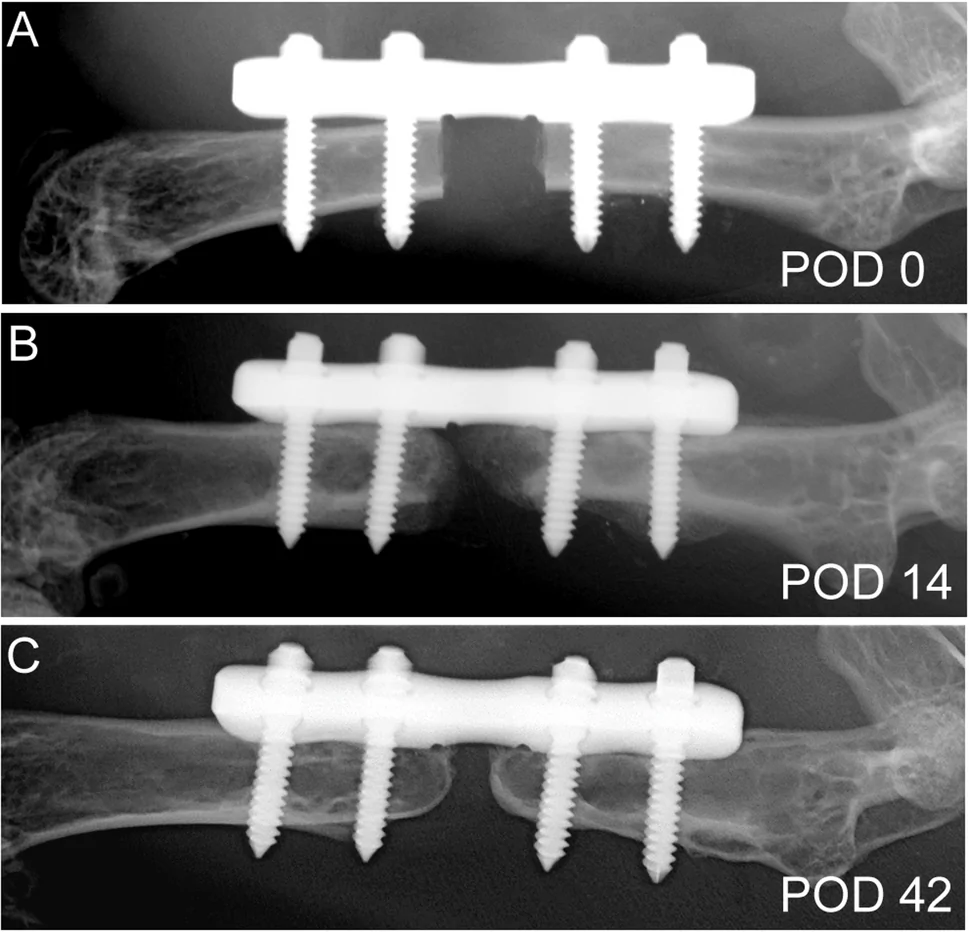
Representative plain films of non-unions. Representative radiographs of 1.75 mm defects at **A** post-operative day (POD) 0, **B** POD 14 (2 weeks), and **C** POD 42 (6 weeks)

Radiographs of 1.75 mm osteotomy femurs at post-operative day (POD) 0 demonstrated the reproducibility of the procedure (Fig. 2A). We observed that new bone formation into the osteotomy gap from both ends of the bone was evident by two weeks (Fig. 2B; with additional examples at POD14 shown in Supplemental Fig. 1). Animals that exhibited radiographic non-union at 6 weeks post-operatively persisted as non-unions radiographically until the end of the study period (Fig. 2C), even when carried out to 12 weeks (Supplemental Fig. 2). There were no discrepancies between observers with regards to which animals were identified as having femoral non-unions based on radiographic criteria.

### Histology

**Fig. 3:**
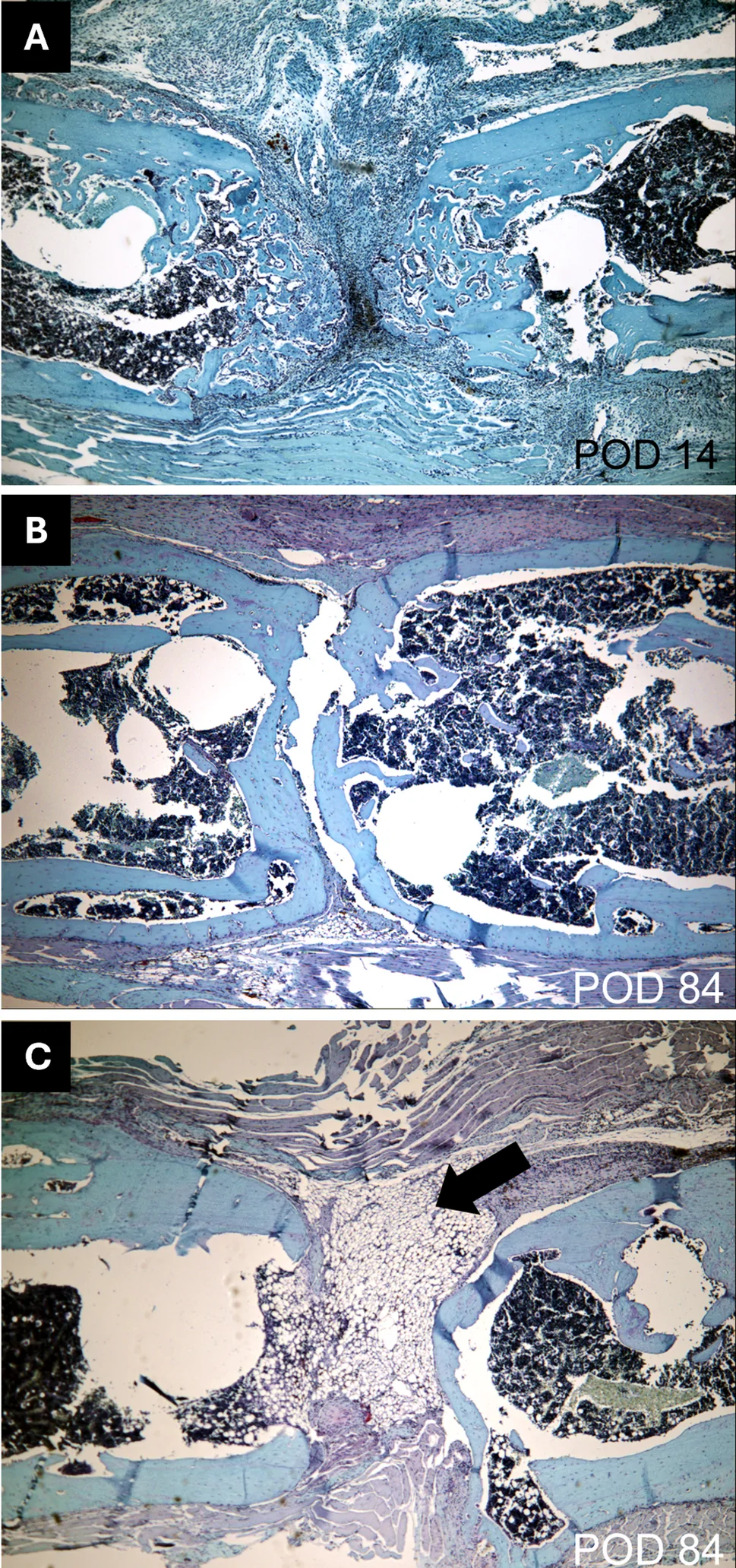
Representative Safranin-O/Fast green histology of bony non-union in 1.75 mm osteotomy model. **A** Images at POD 14 (2 weeks). **B** Additional histology of bony non-union in 1.75 mm osteotomy model at POD 84 (12 weeks), showing bone formation or **C** fibrosis (black arrow) at osteotomy sites

Bone microstructure was examined with Safranin-O/Fast Green staining. By POD 14, extensive periosteal reaction and woven bone formation without bridging was apparent in all 1.75 mm osteotomy animals examined (*n* = 4) (Fig. 3A; see Supplemental Fig. 1 for additional example). Healing was predominantly through intramembranous bone formation, with cartilage observed in one sample (Supplemental Fig. 3, red arrow highlights cartilage formation) which was potentially due to mechanical irritation from the hardware. Otherwise, immunostaining for types I and II collagen confirmed that healing in non-union cases occurred through intramembranous rather than endochondral bone formation (Supplemental Fig. 4, which shows type I collagen immunostaining (top) and type II collagen immunostaining (bottom)). In non-union cases at POD 84, the cut bone ends were covered with bone (Fig. 3B) and/or fibrotic tissue (Fig. 3C), but there was no bridging. In the few cases of union, bony bridging could be seen by POD 42 and the medullary canal was almost completely reestablished by POD 84 (Supplemental Fig. 5).

## Discussion

Many fractures exhibit impaired healing, with up to 100,000 cases progressing to non-union annually [2, 3]. For this reason, there has been a growing interest in biologic therapies that may augment the healing process. For example, the use of genetically modified host cells is one form of biologic therapy that continues to gain interest [26, 30]. One of the best ways to investigate the efficacy of any osteogenic intervention, such as genetically engineered cells, is through a significant biologic challenge such as a critically sized long bone defect in a transgenic mouse model. However, the size of the defect needs to be highly reproducible to enable statistical comparisons.

In this investigation we have demonstrated a model of delayed healing of long bone fractures that manifests as a critical sized defect in approximately 65% of animal subjects. This effect was evident up to 12 weeks post-operatively, which is more than three times the duration to union for smaller defects utilizing the same fixation method [57]. This therefore represents a novel in vivo murine model of delayed long bone healing that can accurately recapitulate human long bone fracture internal fixation. The well-characterized and mutable genome of the mouse gives this model of osteogenesis several significant advantages over other in vivo investigative techniques reported in the literature thus far.

Although many larger animal models have the benefit of utilizing clinically applicable fixation techniques due to their larger osseous elements, these osteogenic models are not amenable to the genetic manipulations that are uniquely possible in the mouse. Altered gene expression in larger animals is usually accomplished by viral transduction of individual cells in vitro. However virally transduced gene products generally exhibit limited ability to integrate into the host genome and thus demonstrate short-lived expression of the desired gene. In contrast to larger mammalian models, there are a large number of genetically altered mice available for investigation as well as a multitude of corresponding reagents available for molecular analysis of fracture repair in mice.

Although other mouse models of osteogenesis exist, they are limited in either their ability to mimic clinically relevant fractures or in their fracture fixation techniques [33–35]. The most common models that implement clinically relevant internal fixation utilized intramedullary fixation, albeit mostly without rotational stability [20, 36, 44, 45, 63]. The resultant assessment of interventions in these models is limited by a less uniform fracture mechanism and the effects of intrinsic variables such as fracture micromotion, which have been shown to affect bone biology and may confound the effects of studied interventions [53, 54]. Furthermore, the overwhelming majority of mice with a closed long bone fracture treated with intramedullary fixation heal. There is additional concern for the clinical applicability of these models given that most murine fracture models heal through endochondral ossification, which may misrepresent clinical fracture healing using modern low strain locked plating internal fixation constructs. Notably, among reported long bone nonunion models in non-transgenic mice, many induce substantial biologic insults or employ less translationally applicable techniques [36]. For example, periosteal stripping is reported to delay callus formation in non-critical defects of mouse femurs [64] but profoundly disrupts biologic progenitors that are prerequisite for intramembranous healing, potentially altering and hindering the efficacy of osteogenic interventions. Other studies report up to a two-fold larger segmental bone defect [59, 65] than the 1.75 mm osteotomy reported in this study. Intramedullary fixation is commonly used [59, 66] and, as described above, introduces associated limitations such as fracture micromotion that may alter bone biology and shift local healing from endochondral to intramembranous ossification [57].

Another advantage of the model used in this investigation is the titanium plate implant used for rigid internal fixation. The titanium locking plate used in this study is directly analogous to plates used for internal fixation in humans, and as such allows for the preservation of periosteal blood supply [34]. Periosteal viability is particularly important when evaluating biologically active therapies, as the periosteum has been proposed to be a source of osteoblast progenitor cells [39, 41, 46, 47, 67]. The internal fixation plate model described here also eliminates the impaired ambulation of other models [68]. The operative animals are able to bear weight without significant distress immediately after the procedure, which we feel demonstrates a more humane mode of fixation than previous reports in the setting of large femoral defects. It should be noted that this model is largely an assessment of intramembranous bony healing and that this is evidenced by the lack of significant cartilage or callous on histologic examination at both 5 days and 2 weeks post-operatively (Fig. 3). Additionally, immunostaining for type II collagen fails to reveal any positive staining at the osteotomy site, while type I collagen demonstrates consistent peripheral staining at the osteotomy site for osteoid and cortical bone (see Data, Supplemental Fig. 4).

Despite the numerous advantages of the model we describe, there are specific weaknesses. Although our locked plating construct is designed to generate a low-strain environment at the osteotomy site, it is possible that the large bone defect allowed for local micromotion in a minority of animals. This may potentially explain the cartilage formation observed in a single femur. However, no other sample demonstrated significant cartilage or callus formation at the osteotomy site, suggesting that this model stimulated intramembranous healing in most cases. Future quantification of construct stiffness and typical femur loads during gait may help to better characterize the local biomechanical environment. Additionally, while most of the femurs receiving a 1.75 mm osteotomy eventually led to non-union, 35% of the animals healed the femoral defect. 1.75 mm is the maximum gap size that can be created with this commercially available locking plate, and future studies to design a locking plate that permits the generation of a larger critical defect are warranted. Biomechanical analyses on the post-surgical femurs after euthanasia would also be warranted. In the present study, the lack of bony healing at the osteotomy site was readily apparent histologically and radiographically. However, larger studies may demonstrate that some of the fractures that achieved visual union are nonetheless mechanically inferior. Another limitation of this specific study is that we were unable to obtain micro-computed tomography scans to provide higher resolution data to supplement our radiographic findings. Despite these limitations, the model presented here enables investigators to create a precisely sized and localized defect to detect healing differences between treatment groups and to account for differences in effect size when calculating a power analysis for proposed investigations.

## Conclusions

In summary, this study describes a non-union model in the mouse femur that primarily demonstrates intramembranous bony healing and utilizes a clinically relevant form of fixation using a locked plating system. As more constructs like this are implemented clinically, models that recapitulate this type of fixation and demonstrate intramembranous healing in a low strain environment will continue to provide translational significance, by serving as a means for gaining clinically relevant data on the biology and genetics of fracture repair. The clinically relevant fixation method, delayed healing, decreased morbidity, and ability to apply this technology to transgenic mice demonstrate the numerous advantages of this model. As a result, this model provides researchers with an excellent pre-clinical fracture biology investigation tool to compassionately assess the contributions of specific genes and osteogenic biologic therapies in vivo.

## Supplementary Information

Below is the link to the electronic supplementary material.

Supplementary Material 1: Additional radiographs and histology of femurs with non-union. Upper and middle panel, additional radiographs of non-unions at POD 14. Lower panel, Safranin-O staining of non-union at POD 14.

Supplementary Material 2: Radiographs at POD0 and POD84 showing typical non-union at POD 84.

Supplementary Material 3: Safranin O staining through non-union at POD 42.

Supplementary Material 4: Immunofluoresence staining for type I collagen (top) and type II collagen (bottom) of a non-union at POD 42.

Supplementary Material 5: Representative radiographic and histologic images of union cases. (A) Plain film of a union at POD 42. (B) Safranin O staining of a union at POD 84.

Supplementary Material 6

## Data Availability

The majority of datasets supporting the conclusions of this article are included within the article and its additional files, with the remaining available from the corresponding author on reasonable request.

## References

[1] Marsell R, Einhorn TA. Emerging bone healing therapies. J Orthop Trauma. 2010;24(Suppl 1):S4–8. 10.1097/BOT.0b013e3181ca3fab 20182234

[2] Einhorn TA. Enhancement of fracture healing. Instr Course Lect. 1996;45:401–16. 8727759

[3] Bishop JA, Palanca AA, Bellino MJ, Lowenberg DW. Assessment of compromised fracture healing. J Am Acad Orthop Surg. 2012;20(5):273–82. 10.5435/JAAOS-20-05-273 22553099

[4] O’Keefe RJ, Mao J. Bone tissue engineering and regeneration: from discovery to the clinic–an overview. Tissue Eng Part B Rev. 2011;17(6):389–92. 10.1089/ten.teb.2011.0475 PMC3223012 21902614

[5] Goldstrohm GL, Mears DC, Swartz WM. The results of 39 fractures complicated by major segmental bone loss and/or leg length discrepancy. J Trauma. 1984;24(1):50–8. 10.1097/00005373-198401000-00008 6363722

[6] Govender S, Csimma C, Genant HK, Valentin-Opran A, Amit Y, Arbel R, et al. Recombinant human bone morphogenetic protein-2 for treatment of open tibial fractures: a prospective, controlled, randomized study of four hundred and fifty patients. J Bone Joint Surg Am. 2002;84(12):2123–34. 10.2106/00004623-200212000-00001 12473698

[7] Johnson EE, Urist MR, Finerman GA. Resistant nonunions and partial or complete segmental defects of long bones. Treatment with implants of a composite of human bone morphogenetic protein (BMP) and autolyzed, antigen-extracted, allogeneic (AAA) bone. Clin Orthop Relat Res. 1992(277):229–37. 1555346

[8] Gazdag AR, Lane JM, Glaser D, Forster RA. Alternatives to Autogenous Bone Graft: Efficacy and Indications. J Am Acad Orthop Surg. 1995;3(1):1–8. 10.5435/00124635-199501000-00001 10790647

[9] Hernigou P, Poignard A, Manicom O, Mathieu G, Rouard H. The use of percutaneous autologous bone marrow transplantation in nonunion and avascular necrosis of bone. J Bone Joint Surg Br. 2005;87(7):896–902. 10.1302/0301-620X.87B7.16289 15972899

[10] Veitch SW, Stroud RM, Toms AD. Compaction bone grafting in tibial plateau fracture fixation. J Trauma. 2010;68(4):980–3. 10.1097/TA.0b013e3181b16e3d 20386289

[11] Nauth A, Miclau T 3rd, Bhandari M, Schemitsch EH. Use of osteobiologics in the management of osteoporotic fractures. J Orthop Trauma. 2011;25(Suppl 2):S51–5. 10.1097/BOT.0b013e31821b8c52 21566475

[12] Enneking WF, Eady JL, Burchardt H. Autogenous cortical bone grafts in the reconstruction of segmental skeletal defects. J Bone Joint Surg Am. 1980;62(7):1039–58. 7000788

[13] Ebraheim NA, Elgafy H, Xu R. Bone-graft harvesting from iliac and fibular donor sites: techniques and complications. J Am Acad Orthop Surg. 2001;9(3):210–8. 10.5435/00124635-200105000-00007 11421578

[14] Backstein D, Safir O, Gross A. Management of bone loss: structural grafts in revision total knee arthroplasty. Clin Orthop Relat Res. 2006;446:104–12. 10.1097/01.blo.0000214426.52206.2c 16672879

[15] Khan SN, Cammisa FP Jr., Sandhu HS, Diwan AD, Girardi FP, Lane JM. The biology of bone grafting. J Am Acad Orthop Surg. 2005;13(1):77–86. 15712985

[16] DeCoster TA, Gehlert RJ, Mikola EA, Pirela-Cruz MA. Management of posttraumatic segmental bone defects. J Am Acad Orthop Surg. 2004;12(1):28–38. 10.5435/00124635-200401000-00005 14753795

[17] Boden SD, Zdeblick TA, Sandhu HS, Heim SE. The use of rhBMP-2 in interbody fusion cages. Definitive evidence of osteoinduction in humans: a preliminary report. Spine (Phila Pa 1976). 2000;25(3):376–81. 10.1097/00007632-200002010-00020 10703113

[18] Hollinger JO, Onikepe AO, MacKrell J, Einhorn T, Bradica G, Lynch S, et al. Accelerated fracture healing in the geriatric, osteoporotic rat with recombinant human platelet-derived growth factor-BB and an injectable beta-tricalcium phosphate/collagen matrix. J Orthop Res. 2008;26(1):83–90. 10.1002/jor.20453 17676626

[19] Einhorn TA, Majeska RJ, Mohaideen A, Kagel EM, Bouxsein ML, Turek TJ, et al. A single percutaneous injection of recombinant human bone morphogenetic protein-2 accelerates fracture repair. J Bone Joint Surg Am. 2003;85(8):1425–35. 10.2106/00004623-200308000-00002 12925621

[20] Hiltunen A, Vuorio E, Aro HT. A standardized experimental fracture in the mouse tibia. J Orthop Res. 1993;11(2):305–12. 10.1002/jor.1100110219 8483044

[21] Aspenberg P, Genant HK, Johansson T, Nino AJ, See K, Krohn K, et al. Teriparatide for acceleration of fracture repair in humans: a prospective, randomized, double-blind study of 102 postmenopausal women with distal radial fractures. J Bone Min Res. 2010;25(2):404–14. 10.1359/jbmr.090731 19594305

[22] Bukata SV. Systemic administration of pharmacological agents and bone repair: what can we expect. Injury. 2011;42(6):605–8. 10.1016/j.injury.2011.03.061 21507402

[23] Carragee EJ, Hurwitz EL, Weiner BK. A critical review of recombinant human bone morphogenetic protein-2 trials in spinal surgery: emerging safety concerns and lessons learned. Spine J. 2011;11(6):471–91. 10.1016/j.spinee.2011.04.023 21729796

[24] Younger EM, Chapman MW. Morbidity at bone graft donor sites. J Orthop Trauma. 1989;3(3):192–5. 10.1097/00005131-198909000-00002 2809818

[25] Subbiah V, Madsen VS, Raymond AK, Benjamin RS, Ludwig JA. Of mice and men: divergent risks of teriparatide-induced osteosarcoma. Osteoporos Int. 2010;21(6):1041–5. 10.1007/s00198-009-1004-0 19597911

[26] Carofino BC, Lieberman JR. Gene therapy applications for fracture-healing. J Bone Joint Surg Am. 2008;90(Suppl 1):99–110. 10.2106/JBJS.G.01546 18292364

[27] Hannallah D, Peterson B, Lieberman JR, Fu FH, Huard J. Gene therapy in orthopaedic surgery. Instr Course Lect. 2003;52:753–68. 12690900

[28] Gamradt SC, Lieberman JR. Genetic modification of stem cells to enhance bone repair. Ann Biomed Eng. 2004;32(1):136–47. 10.1023/b:abme.0000007798.78548.b8 14964729

[29] Hsu WK, Wang JC, Liu NQ, Krenek L, Zuk PA, Hedrick MH, et al. Stem cells from human fat as cellular delivery vehicles in an athymic rat posterolateral spine fusion model. J Bone Joint Surg Am. 2008;90(5):1043–52. 10.2106/JBJS.G.00292 18451397

[30] Hidaka C, Maher S, Packer J, Gasinu S, Cunningham ME, Rodeo S. What’s new in orthopaedic research. J Bone Joint Surg Am. 2009;91(11):2756–70. 10.2106/JBJS.I.00865 19884457

[31] Lieberman JR, Ghivizzani SC, Evans CH. Gene transfer approaches to the healing of bone and cartilage. Mol Ther. 2002;6(2):141–7. 10.1006/mthe.2000.0663 12161179

[32] Jacenko O, Olsen BR. Transgenic mouse models in studies of skeletal disorders. J Rheumatol Suppl. 1995;43:39–41. 7752133

[33] Tay BK, Le AX, Gould SE, Helms JA. Histochemical and molecular analyses of distraction osteogenesis in a mouse model. J Orthop Res. 1998;16(5):636–42. 10.1002/jor.1100160518 9820290

[34] Carvalho RS, Einhorn TA, Lehmann W, Edgar C, Al-Yamani A, Apazidis A, et al. The role of angiogenesis in a murine tibial model of distraction osteogenesis. Bone. 2004;34(5):849–61. 10.1016/j.bone.2003.12.027 15121017

[35] Feeley BT, Conduah AH, Sugiyama O, Krenek L, Chen IS, Lieberman JR. In vivo molecular imaging of adenoviral versus lentiviral gene therapy in two bone formation models. J Orthop Res. 2006;24(8):1709–21. 10.1002/jor.20229 16788987

[36] Gunderson ZJ, Campbell ZR, McKinley TO, Natoli RM, Kacena MA. A comprehensive review of mouse diaphyseal femur fracture models. Injury. 2020;51(7):1439–47. 10.1016/j.injury.2020.04.011 PMC7323889 32362447

[37] Kaneko Y, Minehara H, Sonobe T, Kameda T, Sekiguchi M, Matsushita T, et al. Differences in macrophage expression in induced membranes by fixation method - Masquelet technique using a mouse’s femur critical-sized bone defect model. Injury. 2024;55(6):111135. 10.1016/j.injury.2023.111135 37925281

[38] Sandberg OH, Aspenberg P. Glucocorticoids inhibit shaft fracture healing but not metaphyseal bone regeneration under stable mechanical conditions. Bone Joint Res. 2015;4(10):170–5. 10.1302/2046-3758.410.2000414 PMC4649682 26490971

[39] Morgan EF, Hussein AI, Al-Awadhi BA, Hogan DE, Matsubara H, Al-Alq Z, et al. Vascular development during distraction osteogenesis proceeds by sequential intramuscular arteriogenesis followed by intraosteal angiogenesis. Bone. 2012;51(3):535–45. 10.1016/j.bone.2012.05.008 PMC3412922 22617817

[40] Hu CT, Offley SC, Yaseen Z, O’Keefe RJ, Humphrey CA. Murine model of oligotrophic tibial nonunion. J Orthop Trauma. 2011;25(8):500–5. 10.1097/BOT.0b013e3182249fad 21738067

[41] Colnot C, Thompson Z, Miclau T, Werb Z, Helms JA. Altered fracture repair in the absence of MMP9. Development. 2003;130(17):4123–33. 10.1242/dev.00559 PMC2778064 12874132

[42] Sandberg O, Bernhardsson M, Aspenberg P. Earlier effect of alendronate in mouse metaphyseal versus diaphyseal bone healing. J Orthop Res. 2017;35(4):793–9. 10.1002/jor.23316 27233101

[43] Histing T, Garcia P, Holstein JH, Klein M, Matthys R, Nuetzi R, et al. Small animal bone healing models: standards, tips, and pitfalls results of a consensus meeting. Bone. 2011;49(4):591–9. 10.1016/j.bone.2011.07.007 21782988

[44] Gerstenfeld LC, Sacks DJ, Pelis M, Mason ZD, Graves DT, Barrero M, et al. Comparison of effects of the bisphosphonate alendronate versus the RANKL inhibitor denosumab on murine fracture healing. J Bone Min Res. 2009;24(2):196–208. 10.1359/jbmr.081113 PMC6961532 19016594

[45] Grimes R, Jepsen KJ, Fitch JL, Einhorn TA, Gerstenfeld LC. The transcriptome of fracture healing defines mechanisms of coordination of skeletal and vascular development during endochondral bone formation. J Bone Min Res. 2011;26(11):2597–609. 10.1002/jbmr.486 21826735

[46] Zhang X, Xie C, Lin AS, Ito H, Awad H, Lieberman JR, et al. Periosteal progenitor cell fate in segmental cortical bone graft transplantations: implications for functional tissue engineering. J Bone Min Res. 2005;20(12):2124–37. 10.1359/JBMR.050806 PMC4527562 16294266

[47] Chen D, Shen H, Shao J, Jiang Y, Lu J, He Y, et al. Superior mineralization and neovascularization capacity of adult human metaphyseal periosteum-derived cells for skeletal tissue engineering applications. Int J Mol Med. 2011;27(5):707–13. 10.3892/ijmm.2011.634 21369695

[48] Jang Y, Lopas LA, Foley DP, McKinley TO, Mullis BH, Collier CD, et al. Of Mice and Men: Temporal Comparison of Femoral Shaft Fracture Healing After Intramedullary Nailing: Retrospective Observational Study of Modified Radiographic Union Scores for Tibia. J Bone Joint Surg Am. 2025;107(16):1841–7. 10.2106/JBJS.24.01304 PMC12344732 40833385

[49] Manigrasso MB, O’Connor JP. Characterization of a closed femur fracture model in mice. J Orthop Trauma. 2004;18(10):687–95. 10.1097/00005131-200411000-00006 15507822

[50] Zwingenberger S, Niederlohmann E, Vater C, Rammelt S, Matthys R, Bernhardt R, et al. Establishment of a femoral critical-size bone defect model in immunodeficient mice. J Surg Res. 2013;181(1):e7–14. 10.1016/j.jss.2012.06.039 PMC4045639 22765996

[51] Hixon KR, McKenzie JA, Sykes DAW, Yoneda S, Hensley A, Buettmann EG, et al. Ablation of Proliferating Osteoblast Lineage Cells After Fracture Leads to Atrophic Nonunion in a Mouse Model. J Bone Min Res. 2021;36(11):2243–57. 10.1002/jbmr.4424 PMC8719642 34405443

[52] Shoji S, Uchida K, Satio W, Sekiguchi H, Inoue G, Miyagi M, et al. Acceleration of bone union by in situ-formed hydrogel containing bone morphogenetic protein-2 in a mouse refractory fracture model. J Orthop Surg Res. 2020;15(1):426. 10.1186/s13018-020-01953-7 PMC7501615 32948214

[53] Palomares KT, Gleason RE, Mason ZD, Cullinane DM, Einhorn TA, Gerstenfeld LC, et al. Mechanical stimulation alters tissue differentiation and molecular expression during bone healing. J Orthop Res. 2009;27(9):1123–32. 10.1002/jor.20863 PMC2726267 19242967

[54] Moukoko D, Pourquier D, Pithioux M, Chabrand P. Influence of cyclic bending loading on in vivo skeletal tissue regeneration from periosteal origin. Orthop Traumatol Surg Res. 2010;96(8):833–9. 10.1016/j.otsr.2010.07.006 21036687

[55] Claes LE, Heigele CA. Magnitudes of local stress and strain along bony surfaces predict the course and type of fracture healing. J Biomech. 1999;32(3):255–66. 10.1016/s0021-9290(98)00153-5 10093025

[56] Claes L, Eckert-Hubner K, Augat P. The fracture gap size influences the local vascularization and tissue differentiation in callus healing. Langenbecks Arch Surg. 2003;388(5):316–22. 10.1007/s00423-003-0396-0 13680236

[57] Histing T, Garcia P, Matthys R, Leidinger M, Holstein JH, Kristen A, et al. An internal locking plate to study intramembranous bone healing in a mouse femur fracture model. J Orthop Res. 2010;28(3):397–402. 10.1002/jor.21008 19780192

[58] Manassero M, Decambron A, Huu Thong BT, Viateau V, Bensidhoum M, Petite H. Establishment of a Segmental Femoral Critical-size Defect Model in Mice Stabilized by Plate Osteosynthesis. J Vis Exp. 2016(116). 10.3791/52940 PMC5092194 27768070

[59] Kaur A, Mohan S, Rundle CH. A segmental defect adaptation of the mouse closed femur fracture model for the analysis of severely impaired bone healing. Anim Model Exp Med. 2020;3(2):130–9. 10.1002/ame2.12114 PMC7323699 32613172

[60] Srouji S, Ben-David D, Kohler T, Muller R, Zussman E, Livne E. A model for tissue engineering applications: femoral critical size defect in immunodeficient mice. Tissue Eng Part C Methods. 2011;17(5):597–606. 10.1089/ten.TEC.2010.0501 21254818

[61] Retting KN, Song B, Yoon BS, Lyons KM. BMP canonical Smad signaling through Smad1 and Smad5 is required for endochondral bone formation. Development. 2009;136(7):1093–104. 10.1242/dev.029926 PMC2668702 19224984

[62] DiSilvio F Jr., Foyil S, Schiffman B, Bernstein M, Summers H, Lack WD. Long Bone Union Accurately Predicted by Cortical Bridging within 4 Months. JB JS Open Access. 2018;3(4):e0012. 10.2106/JBJS.OA.18.00012 PMC6400511 30882051

[63] Bonnarens F, Einhorn TA. Production of a standard closed fracture in laboratory animal bone. J Orthop Res. 1984;2(1):97–101. 10.1002/jor.1100020115 6491805

[64] Grongroft I, Wissing S, Meesters DM, Poeze M, Matthys-Mark R, Ito K, et al. Development of a novel murine delayed secondary fracture healing in vivo model using periosteal cauterization. Arch Orthop Trauma Surg. 2019;139(12):1743–53. 10.1007/s00402-019-03255-y PMC6825648 31399754

[65] Manassero M, Viateau V, Matthys R, Deschepper M, Vallefuoco R, Bensidhoum M, et al. A novel murine femoral segmental critical-sized defect model stabilized by plate osteosynthesis for bone tissue engineering purposes. Tissue Eng Part C Methods. 2013;19(4):271–80. 10.1089/ten.TEC.2012.0256 22953787

[66] Menger MM, Bauer D, Bleimehl M, Scheuer C, Ehnert S, Menger MD, et al. Comparison of two non-union models with damaged periosteum in mice: Segmental defect and pin-clip fixation versus transverse fracture and K-wire stabilization. Bone. 2022;162:116475. 10.1016/j.bone.2022.116475 35752408

[67] Ball MD, Bonzani IC, Bovis MJ, Williams A, Stevens MM. Human periosteum is a source of cells for orthopaedic tissue engineering: a pilot study. Clin Orthop Relat Res. 2011;469(11):3085–93. 10.1007/s11999-011-1895-x PMC3183217 21547415

[68] Histing T, Kristen A, Roth C, Holstein JH, Garcia P, Matthys R, et al. In vivo gait analysis in a mouse femur fracture model. J Biomech. 2010;43(16):3240–3. 10.1016/j.jbiomech.2010.07.019 20832805

