# Supplementary material for: A mouse model of non-union in a femur critical-size defect using locking plate fixation
Source: J Orthop Surg Res. 2026 Jul 23;21:625. doi: 10.1186/s13018-026-07099-2 (PMC13737778; doi:10.1186/s13018-026-07099-2)
Supplement: Supplementary file 1 [file 13018_2026_7099_MOESM6_ESM.docx]

**Supplemental Tables and Legends**

**Supplemental Table 1: Radiographic and histologic classification of animals treated with 0.25mm osteotomy and euthanized at 6 weeks or 12 weeks post-operatively**

| **Sample ID** | **Osteotomy Size** | **Duration from surgery to Euthanasia** | **Combined Radiographic/ Histologic Classification** | **Radiographic/ Histologic Assessment by Observer #1** | **Radiographic/ Histologic Assessment by Observer #2** | **Radiographic/ Histologic Assessment by Observer #3** |
| --- | --- | --- | --- | --- | --- | --- |
| 3916 | 0.25mm | 12 weeks | Union/Union | Union/Union | Union/Union | Union/Union |
| 3952 | 0.25mm | 6 weeks | Union/Union | Union/Union | Union/Union | Union/Union |
| 3955 | 0.25mm | 6 weeks | Union/Union | Union/Union | Union/Union | Union/Union |
| 3957 | 0.25mm | 6 weeks | Union/Union | Union/Union | Union/Union | Union/Union |

**Supplemental Table 2: Radiographic and histologic classification of animals treated with 1.75mm osteotomy and euthanized at 2 weeks post-operatively**

| **Sample ID** | **Osteotomy Size** | **Combined Radiographic/ Histologic Classification** | **Radiographic/ Histologic Assessment by Observer #1** | **Radiographic/ Histologic Assessment by Observer #2** | **Radiographic/ Histologic Assessment by Observer #3** |
| --- | --- | --- | --- | --- | --- |
| 5722 | 1.75mm | Nonunion | Nonunion / Nonunion | Nonunion / Nonunion | Nonunion / Nonunion |
| 5723 | 1.75mm | Nonunion | Nonunion / Nonunion | Nonunion / Nonunion | Nonunion / Nonunion |
| 5724 | 1.75mm | Nonunion | Nonunion / Nonunion | Nonunion / Nonunion | Nonunion / Nonunion |
| 5725 | 1.75mm | Nonunion | Nonunion / Nonunion | Nonunion / Nonunion | Nonunion / Nonunion |

**Supplemental Table 3: Radiographic and histologic classification of animals treated with 1.75mm osteotomy and euthanized at 6 weeks post-operatively**

| **Sample ID** | **Osteotomy Size** | **Combined Radiographic/ Histologic Classification** | **Radiographic/ Histologic Assessment by Observer #1** | **Radiographic/ Histologic Assessment by Observer #2** | **Radiographic/ Histologic Assessment by Observer #3** |
| --- | --- | --- | --- | --- | --- |
| 3953 | 1.75mm | Nonunion | Nonunion/ Nonunion | Nonunion/ Nonunion | Nonunion/ Nonunion |
| 3954 | 1.75mm | Nonunion | Nonunion/ Nonunion | Nonunion/ Nonunion | Nonunion/ Nonunion |
| 5684 | 1.75mm | Nonunion | Nonunion/ Nonunion | Nonunion/ Nonunion | Nonunion/ Nonunion |
| 5685 | 1.75mm | Union | Union/Union | Union/Union | Union/Union |
| 5690* | 1.75mm | Nonunion | Nonunion/ Nonunion | Nonunion/ Nonunion | Nonunion/ Nonunion |
| 5692 | 1.75mm | Union | Union/Union | Union/Union | Union/Union |
| 5693 | 1.75mm | Nonunion | Nonunion/ Nonunion | Union/ Nonunion | Union/ Nonunion |

* = samples pictured in figures

**Supplemental Table 4: Radiographic and histologic classification of animals treated with 1.75mm osteotomy and euthanized at 12 weeks post-operatively**

| **Sample ID** | **Osteotomy Size** | **Combined Radiographic/ Histologic Classification** | **Radiographic/ Histologic Assessment by Observer #1** | **Radiographic/ Histologic Assessment by Observer #2** | **Radiographic/ Histologic Assessment by Observer #3** |
| --- | --- | --- | --- | --- | --- |
| 3915 | 1.75mm | Nonunion | Nonunion/ Nonunion | Nonunion/ Nonunion | Nonunion/ Nonunion |
| 5679 | 1.75mm | Union | Union/Union | Union/Union | Union/Union |
| 5680 | 1.75mm | Union | Union/Union | Union/Union | Union/Union |
| 5701 | 1.75mm | Nonunion | Nonunion/ Nonunion | Nonunion/ Nonunion | Nonunion/ Nonunion |
| 5702 | 1.75mm | Nonunion | Nonunion/ Nonunion | Nonunion/ Nonunion | Nonunion/ Nonunion |
| 5703 | 1.75mm | Nonunion | Nonunion/ Nonunion | Nonunion/ Nonunion | Nonunion/ Nonunion |
| 5704 | 1.75mm | Union | Union/Union | Union/Union | Union/Union |
| 5705 | 1.75mm | Nonunion | Nonunion/ Nonunion | Union/ Nonunion | Nonunion/ Nonunion |
| 5707* | 1.75mm | Nonunion | Nonunion/ Nonunion | Union/ Nonunion | Nonunion/ Nonunion |
| 5708 | 1.75mm | Union | Union/Union | Union/Union | Union/Union |
| 5709 | 1.75mm | Union | Union/Union | Union/Union | Union/Union |
| 5710 | 1.75mm | Non-union | Nonunion/ Nonunion | Union/ Nonunion | Nonunion/ Nonunion |
| 5711^#^ | 1.75mm | Union | Union/Union | Union/Union | Union/Union |

* = samples pictured in figures

\# = excluded from analysis because loss of fixation at distal screw
